# Proteomic and Biological Analysis of an *In Vitro* Human Endothelial System in Response to Drug Anaphylaxis

**DOI:** 10.3389/fimmu.2021.692569

**Published:** 2021-06-25

**Authors:** Alma Yuste-Montalvo, Sergio Fernandez-Bravo, Tamara Oliva, Carlos Pastor-Vargas, Diana Betancor, María José Goikoetxea, José Julio Laguna, Juan Antonio López, Gloria Alvarez-Llamas, Javier Cuesta-Herranz, Marta Martin-Lorenzo, Vanesa Esteban

**Affiliations:** ^1^ Allergy and Inmunology Department, Instituto de Investigaciones Sanitarias (ISS)-Fundación Jiménez Díaz, Universidad Autónoma de Madrid (UAM), Madrid, Spain; ^2^ Red de ASMA, REACCIONES ADVERSAS Y ALÉRGICAS (ARADyAL), Instituto de Salud Carlos III, Madrid, Spain; ^3^ Department of Biochemistry and Molecular Biology, Universidad Complutense de Madrid, Madrid, Spain; ^4^ Department of Allergy, Hospital Universitario Fundación Jiménez Díaz, Madrid, Spain; ^5^ Department of Allergy and Clinic Immunology, IdiSNA, Clínica Universidad de Navarra, Pamplona, Spain; ^6^ Allergy Unit, Allergo-Anaesthesia Unit, Hospital Central de la Cruz Roja, Madrid, Spain; ^7^ Faculty of Medicine and Biomedicine, Alfonso X El Sabio University, Madrid, Spain; ^8^ Cardiovascular Proteomics Laboratory, Centro Nacional de Investigaciones Cardiovasculares (CNIC), Madrid, Spain; ^9^ Inmunoallergy and Proteomics Laboratory, Instituto de Investigaciones Sanitarias (ISS)-Fundación Jiménez Díaz, UAM, Madrid, Spain; ^10^ Red de Investigación Renal (REDINREN), Instituto de Salud Carlos III, Madrid, Spain

**Keywords:** endothelium, anaphylaxis, proteomics, coagulation and complement systems, drug allergy, system biological analysis

## Abstract

Anaphylaxis is a life-threatening systemic hypersensitivity reaction. During anaphylaxis, mediator release by effector cells causes endothelial barrier breakdown, increasing vascular permeability and leakage of fluids, which may lead to tissue edema. Although endothelial cells (ECs) are key players in this context, scant attention has been paid to the molecular analysis of the vascular system, and further analyses of this cell type are necessary, especially in humans. The protein expression pattern of human microvascular ECs was analyzed in response to sera from anaphylactic patients (EC-anaphylaxis) and sera from non-allergic subjects (EC-control) after 2 hours of contact. Firstly, a differential quantitative proteomic analysis of the protein extracts was performed by mass spectrometry using an isobaric labeling method. Second, the coordinated behavior of the identified proteins was analyzed using systems biology analysis (SBA). The proteome of the EC-anaphylaxis system showed 7,707 proteins, of which 1,069 were found to be significantly altered between the EC-control and EC-anaphylaxis groups (p-value < 0.05). Among them, a subproteome of 47 proteins presented a high rate of change (|ΔZq| ≥ 3). This panel offers an endothelial snapshot of the anaphylactic reaction. Those proteins with the highest individual changes in abundance were hemoglobin subunits and structural support proteins. The interacting network analysis of this altered subproteome revealed that the coagulation and complement systems are the main biological processes altered in the EC-anaphylactic system. The comprehensive SBA resulted in 5,512 functional subcategories (biological processes), 57 of which were significantly altered between EC-control and EC-anaphylaxis. The complement system, once again, was observed as the main process altered in the EC system created with serum from anaphylactic patients. Findings of the current study further our understanding of the underlying pathophysiological mechanisms operating in anaphylactic reactions. New target proteins and relevant signaling pathways operating in the *in vitro* endothelial-serum system have been identified. Interestingly, our results offer a protein overview of the micro-EC-anaphylaxis environment. The relevance of the coagulation, fibrinolytic, contact and complement systems in human anaphylaxis is described. Additionally, the untargeted high-throughput analysis used here is a novel approach that reveals new pathways in the study of the endothelial niche in anaphylaxis.

## Introduction

Anaphylaxis is defined as a serious systemic hypersensitivity reaction that is usually rapid onset and may be lethal. Severe anaphylaxis is characterized by potentially life-threatening compromise in the airway, breathing, and/or circulation, and may occur without presence of typical skin features or circulatory shock (1). There is compelling evidence of a global increase in anaphylaxis rates in recent years (2). Several epidemiological studies show that drug-induced anaphylaxis (DIA) has increased dramatically in the last decade (3, 4). Indeed, DIA is one of the leading causes of fatal anaphylaxis in adults, the most common triggers being antibiotics and non-steroidal anti-inflammatory drugs (NSAID) (5, 6). Mechanistically, facing an antigen exposition, Th lymphocytes polarize to Th2 which help in the isotype change of lymphocytes B producing Immunoglobulin E (IgE). Successive antigen contacts induce the cross-linking of IgE molecules with FcϵRI receptors in effector cells (mast cells and basophils) activating them and releasing anaphylactic mediators (7). DIA are classified according to the underlying molecular mechanisms (8). Since IgE-mediated anaphylaxis is the major immune mechanism of allergic anaphylaxis, there are also other immunological mechanisms, especially via IgG. Moreover, non-immune molecular pathways through mediator release or activation of plasma-protein system have been described (1, 9). One such mechanisms is the activation of the cascade of proteins belonging to the complement system (10).

According to the most up-to-date definition of anaphylaxis, the involvement of the vascular system is of high relevance and treatments are aimed at controlling the cardiovascular system when these reactions occur (8, 11, 12). Hypotension and hypoxia are determining factors in anaphylaxis severity, and involvement by the cardiovascular and respiratory systems makes these reactions life-threatening (12, 13). In fact, some reactions are characterized by the presence of edema in the upper respiratory tract and circulatory collapse (14, 15).

Vascular permeability is mainly regulated by the endothelium, and alterations to the endothelium can be harmful and associated with anaphylaxis (16). In humans, 35 years ago a clinical case described “loss of up to 35% of intravascular fluid to the extravascular space within the first 10 minutes of the anaphylactic reaction” (17). At the molecular and cellular level, the action of the mediators released during anaphylactic reactions causes a sudden increase in vascular permeability. Moreover, mediator actions lead to the relaxation of vascular smooth muscle and contraction of the bronchial smooth muscle (18). Functional interaction between endothelial cells (ECs) and vascular smooth muscle cells (VSMCs) regulate vascular resistance. Vasodilation, understood as the loss of vascular resistance, is a clear sign of homeostatic dysregulation. Importantly, different authors attribute a role in anaphylaxis to the endothelial nitric oxide synthase and the release of nitric oxide (NO) from ECs (19, 20). Therefore, both endothelial and VSMCs merit greater attention, as they may lead to new insights into anaphylaxis (21).

The endothelial barrier is a monolayer in charge of the physical boundary between blood and tissues. It contains ECs whose main function is to preserve vascular homeostasis while maintaining the integrity of the barrier (22). Therefore, ECs confer a functional plasticity to adapt to physiological stressors and supply defined angiocrine factors that contribute to the metabolic homeostasis of other organs (23, 24). Some of these multiple substances that ECs produce and release participate in vascular regulation, as well as, coagulation and fibrinolysis processes (25). In its baseline state, the endothelium behaves as a selective layer that regulates the homeostatic balance, controlling the exchange of fluids, solutes, and cells with the nearby tissue. In response to inflammatory or vasoactive stimuli, the endothelium is activated, thereby producing an increase of vascular permeability mainly through the transcellular and paracellular routes (26–29).

Since it is clear that the endothelial niche modulates key processes in anaphylaxis and that there is a substantial rise in DIA, we aim to study EC behavior in response to serum from human drug-anaphylactic and control subjects by using an *in vitro* system and a proteomic approach. We applied a non-biased (non-target) quantitative approach based on peptide labeling tandem mass tag (TMT). Additionally, we performed a network analysis of the proteome-based identified molecules and carried out a systems biology study of the coordinated behavior of those identified proteins to search for main biological processes involved in the endothelial response to anaphylaxis.

## Materials and Methods

### Patient Selection and Samples Collection

The recruitment of patients and collection of sera derived from patients with anaphylaxis were carried out in the emergency departments of two hospitals (Hospital Universitario Fundación Jiménez Díaz, Madrid, Spain and Clínica Universidad de Navarra, Pamplona, Spain), both included in the Asthma, Adverse Drug Reactions and Allergy (ARADyAL) network.

In accordance with the Declaration of Helsinki as regards the collection and processing of biological samples of peripheral blood, the protocols were approved by the IIS-FJD Clinical Research Ethics Committee (PIC38/2016, PIC142/016, and PIC057-19). Subjects of interest were informed of the purpose of the research and provided signed consent to participate. Patients were diagnosed with anaphylaxis by allergists according to the diagnostic criteria published by the World Allergy Organization (WAO) and subsequently the European Academy of Allergy and Clinical Immunology (EAACI) (30, 31). Healthy non-allergic volunteers consisting of subjects who had a negative result for immediate hypersensitivity on skin prick test for the most common allergens were used as controls (32). Anaphylactic samples were collected in serum vacutainers within the first 2 hours as of the onset of symptoms. Anaphylactic and control sera were obtained by centrifugation (3000 g for 10 minutes at 4°C), aliquoted and stored at -80°C until further analysis.

The samples used in our studies were selected based on the severity of the anaphylactic reactions according to the signs and symptoms indicated in the Delphi methodology. Five severity grades have been established through mild to severe reactions. Grades 1 and 2 include mostly skin, gastrointestinal and mucosal/angioedema affectation while those grades from 3 to 5 include cardiovascular, neurologic and respiratory clinical criteria. Based on that, four out of the five patient samples used were moderate reactions classified as grade 4 accordingly to the severity grading system recently stated by Sampson et al. (33). The diagnosis was supported through serum tryptase (ST) measurement, the mean was 28.7 µg/L (maximum 48 µg/L; minimum 11 µg/L) (34). Drugs (antibiotics or NSAID) were identified as the triggers of all reactions. In the case of serum samples from control subjects, the measured ST values ​​were less than 5 µg/l. The clinical characteristics of the sera from DIA patients are shown in **Table 1**.

**Table 1:** Clinical features of the anaphylactic reaction sera.

Sex	Age	Clinical manifestations						Grade of Severity	ST (µg/L)	Trigger	Medication					
		Skin System	Digestive System	Respiratory System	Cardiovascular System	HR (bpm)	SpO_2_				ADRENALINE	ANTI-H1	ANTI-H2	CTC	Personal History
F	76	**+**	**+**	**+**	**+** (HP)	76	93	4	48	NS		**+**		**+**	HT, HU, OA
F	36	**+**		**+**		117	80	4	38.6	AB					AS, OA
F	46	**+**		**+**	**+** (HP)			4	27.2	NS	**+**	**+**	**+**	**+**	HT, DA
F	58				**+** (HP)	125	68	4	18.7	AB	**+**	**+**		**+**	FA, HT, DA, O, OSAHS
M	48	**+**		**+**		98	95	3	11	NS	**+**	**+**		**+**	AS, FA, AD, RC, S

M, male; F, female; HP, hypotension; HR; heart rate; bpm (beats per minute); SpO_2_, percent oxygen saturation; Grade of severity (4 or 3); ST, serum tryptase; NS, nonsteroidal anti-inflammatory drugs (NSAIDs); AB, antibiotics; ANTI-H1, antihistaminic H1; ANTI-H2, antihistaminic H2; CTC, corticosteroids; HT, hypertension, HU, hyperuricemia; PA, previous anaphylactic events; AS, asthma; FA, food allergy, DA, drug allergy; O, obesity; OSAHS, obstructive sleep apnea-hypopnea syndrome; AD, atopic dermatitis; RC, rhinoconjunctivitis; S, smoker.

### *In Vitro* Human Cell Cultures, Serum Incubation, and Protein Extraction

**Figure 1:**
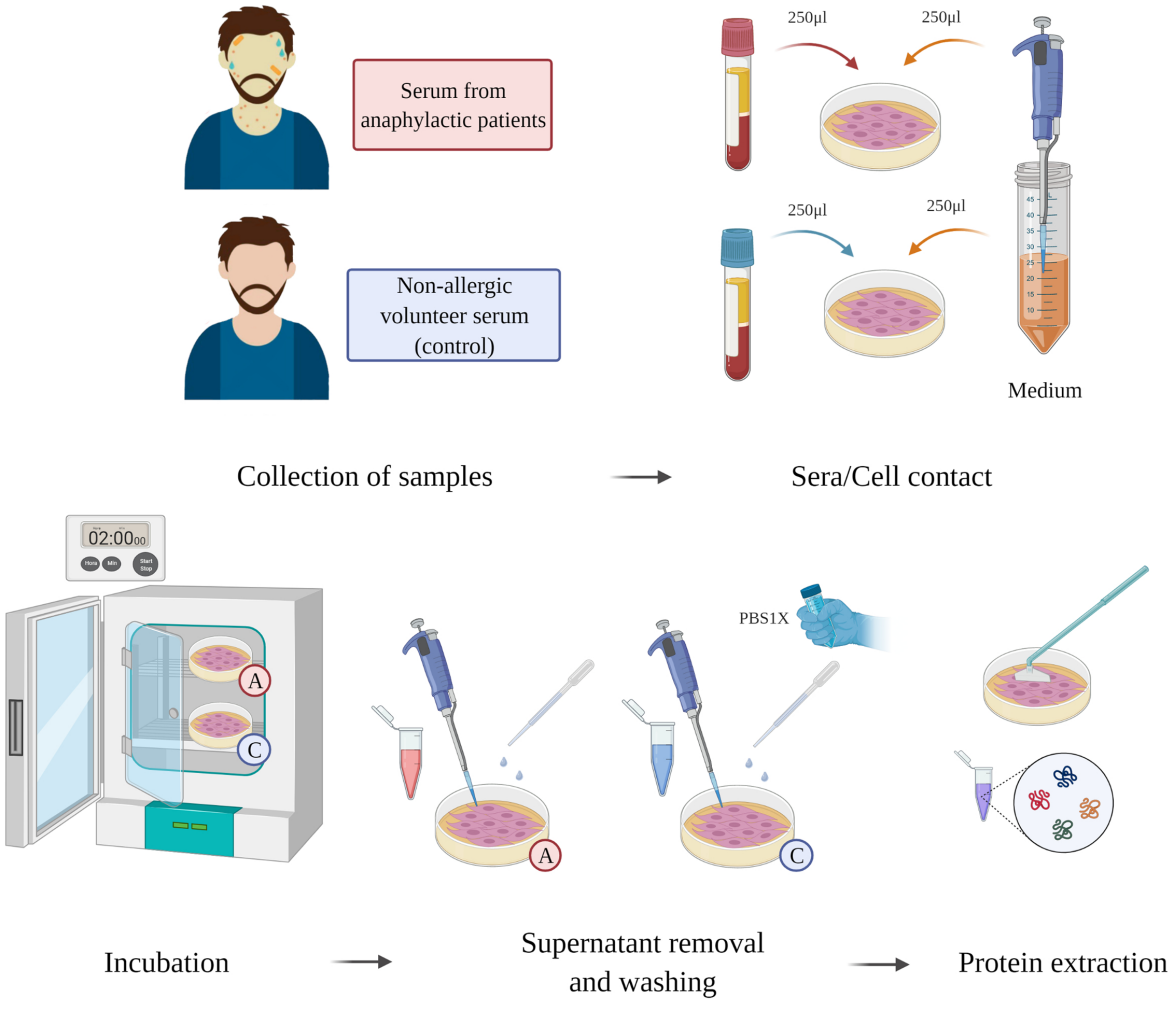
Sera-EC systems workflow. ECs were incubated together sera from anaphylactic patients (n = 5) or sera from control subjects (n = 5) during for 2 hours. Supernatant was then removed; the ECs were washed and then an ECs protein extraction was performed.

Human lung microvascular ECs (CC-2527, Lonza) were used following the protocols provided by the manufacturer. The cells were grown and maintained with EGM-2 medium in combination with the recommended supplements for microvascular cells (CC-3202, Lonza). ECs were seeded in 60-mm polystyrene plates (Corning, Cultek) indicated for the cultivation of adherent cells until monolayer formation. Once serum bovine fetal depletion was completed for 18 hours at 0.5%, sera from anaphylactic or control patients were incubated together with the endothelial monolayers (EC-anaphylaxis and EC-control respectively). The protocol is summarized graphically in **Figure 1**. The protocol was carried out at 1:1 ratio of patient serum/EGM-2 medium non supplemented. After 2 hours of contact, the incubations were removed and the cells were washed 5 times with cold PBS. Next, using a scraper, ECs were lifted from the surface of the plates and lysed under constant agitation with a lysis buffer composed of Tris-HCl (50 mM), DTT (10 mM), and 2% SDS (Bio-rad) (for 30 minutes at 4°C). A bath-sonication step was carried out for 5 minutes to reduce DNA viscosity and centrifugation at 13800 g was performed for 15 minutes. Finally, the protein extracts were stored at -20°C until further analysis.

### Protein Digestion and Isobaric Labeling: Peptide Labeling Using TMT10-Plex

Protein extracts were digested following the Filter-Aided Simple Preparation method with slight modifications (35). Firstly, the protein extracts were diluted in 50 mM TrisHCl pH8, 8 M Urea, and 2 mM Tris (2-carboxyethyl) phosphine (TU buffer). Secondly, iodoacetamide was added to 20 mM in TU buffer for alkylation. After incubation in the dark at room temperature (RT) for 20 minutes, the samples were loaded onto PALL filters (Pall Life Sciences) with a cut-off point of 30 KDa. Then, they were washed three times with TU and another three times with 50 mM ammonium bicarbonate. For the digestion of the proteins retained in the filter, trypsin resuspended in 50 mM ammonium bicarbonate was added at a 1:40 ratio (trypsin:protein) and incubated at 37°C overnight. The resulting peptides were obtained by centrifugation, followed by another centrifugation with a 0.5-M NaCl solution. After that, the peptides were acidified with trifluoroacetic acid (TFA) to 1% and desalted on OASIS HLB columns (Waters) following the manufacturer’s protocol. Finally, the peptides eluted from the column were dried with a Savant vacuum centrifuge (ThermoScientific). Each digested sample was resuspended in 150 mM triethylammonium bicarbonate buffer and labeled for 1 hour at RT with a TMT isobaric labeling reagent (TMT-10plex™ kit, Fisher Scientific) resuspended in anhydrous acetonitrile (ACN). To stop the reaction, each sample was acidified with 25% TFA and incubated for 15 minutes at RT, before mixing all the samples in a single tube. The labeled peptide mixture was concentrated in vacuo on a Speed ​​Vac to evaporate any ACN residues. Unbound material was removed as before on OASIS HLB columns and dried in vacuo. The samples were stored at -20°C for subsequent analysis and fractionation.

### Protein Identification and Quantification by LC-MS/MS

To increase the number of identified peptides, the labeled peptide mixtures were resuspended in 0.2% TFA and 2% ACN, sonicated for 10 minutes, and the peptides were fractionated on high pH C18 reversed phase columns (High pH Fractionation Kit; Thermo Scientific) following the supplier’s protocol. Once the samples were dry, they were stored at -20°C until analysis by liquid chromatography coupled to mass spectrometry (LC-MS/MS). High-resolution analyses of the TMT-labeled peptides were performed on an Easy nLC 1000 nanoHPLC chromatography (Thermo Fisher Scientific) coupled to an Orbitrap Fusion Tribrid mass spectrometer (Thermo Fisher Scientific). The peptides, resuspended in formic acid (FA), were loaded onto a pre-column (PepMap100 C18 LC 75 µm ID, 2 cm, Thermo Scientific) and separated on-line on a NanoViper PepMap100 C18 LC analytical column (75 µm ID, 50 cm, Thermo Scientific) in a continuous 90% ACN gradient; 0.1% FA of 8-31% for 240 minutes and 31-90% for 2 minutes with a flow of 200 nL/minutes.

**Figure 2:**
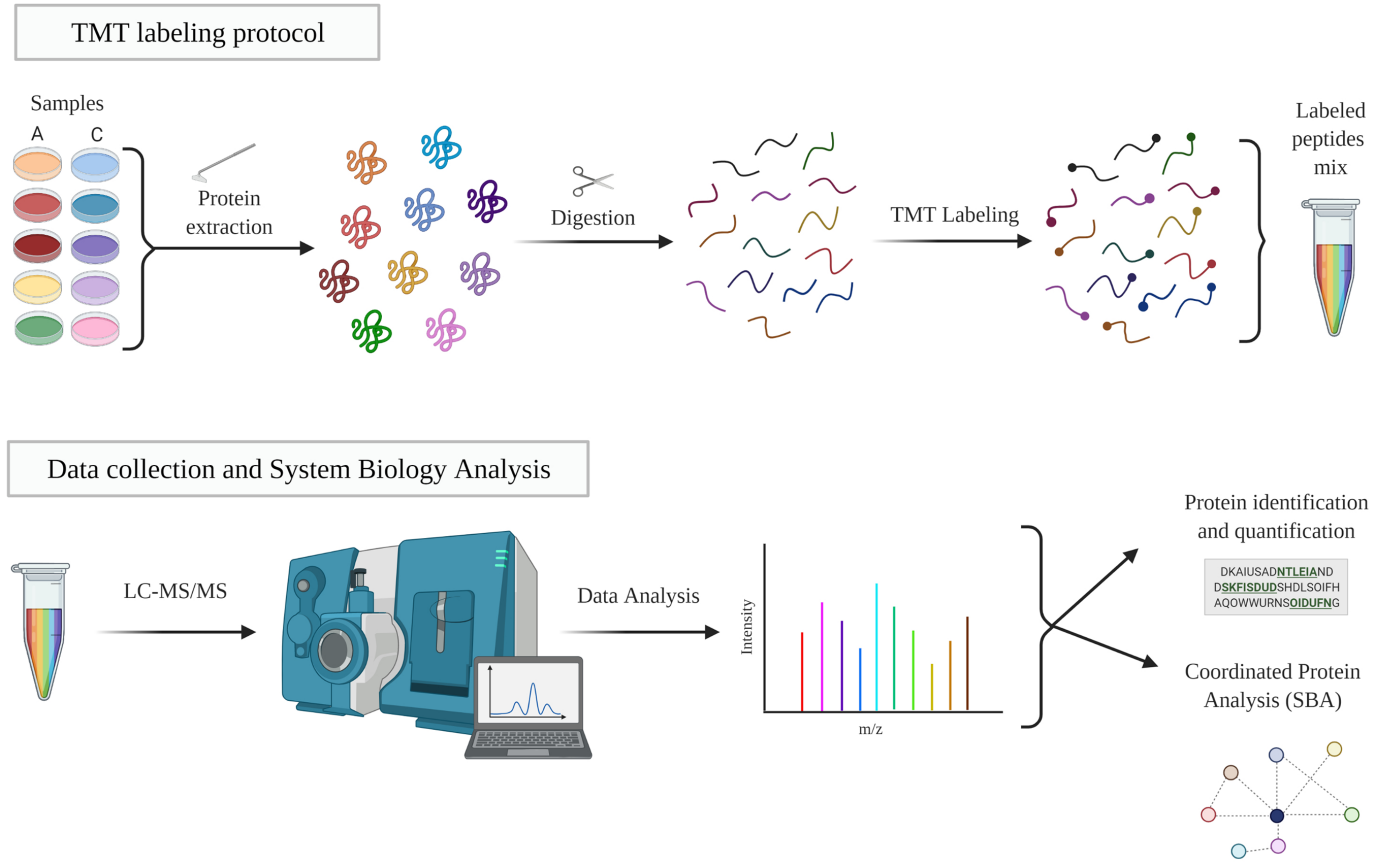
TMT workflow. Protein extracts were digested, labeled and analyzed by LC-MS/MS. The resulting proteins were identified and quantified and then studied individually and collectively studied. A, EC-anaphylaxis; C, EC-control; LC-MS/MS, liquid chromatography-mass spectrometry.

For peptide identification, the fragmentation spectra were analyzed using the SEQUEST HT algorithm in the Proteome Discoverer 2.1 program (Thermo Fisher Scientific). Sequence analysis was carried out by matching the experimental fragmentation spectra with the theoretical spectra from the uniprot.org database (homo sapiens at Nov 2016 70902 sequences). The identification of the peptides was validated with the probability ratio method (36), and the false discovery rate (FDR) of peptide identification was calculated taking into account the search results in the UniProt database with respect to those of a decoy database, according to the previously described method (37). Only those peptides with an FDR less than 1% were considered as having been identified. The protocol is summarized in **Figure 2**.

Quantification of the reporter ions derived from the isobaric labeling of the fragmentation spectra was performed with the in-house software SanXoT (38). Data were analyzed using a logarithmic statistical model (log2), which can estimate the abundances of the peptides and proteins (Zq) from which these reporter ions are derived (WSPP, Weighted Scan Peptide and Protein). The selected proteins were those identified with a number of peptides (Np) Np ≥ 3 that included a log p value (-log p) of between -1.5 and 1.5 and, in particular, those with a -3 ≥ Zq mean ≥ 3 were defined as the selection criteria.

### Data Analysis and Visualization

The scattering of the data between the study groups was carried out by principal component analysis (PCA). The PCA was generated using the Clustvis web tool (link: https://biit.cs.ut.ee/clustvis/). The representation of the identified proteome as well as the heatmap were made using Python 3 and Matplotlib.

### Interaction Network Analysis (STRING)

Interaction networks involving the subproteome composed of those proteins selected with the previous criteria were also investigated. The STRING v11 platform (Search Tool for Retrieval of Interacting Genes/Proteins, https://string- db.org) was used based on known (database and experimentally determined), predicted (gene related), and other (text extraction, protein co-expression, and homology) interactions. To facilitate visualization, nodes disconnected from the network were hidden. For the analysis, the minimum required interaction score was also set with the highest confidence (0.900) and the MLC grouping tool was used (39).

### Systems Biology Analysis

A System Biology Analysis (SBA) of the whole set of identified proteins was performed using a novel algorithm developed specifically for the analysis of coordinated protein responses in high-throughput quantitative proteomics experiments as published elsewhere (40). In line with the quantitative analysis, the Systems Biology Triangle (SBT) model, based on the WSPP model, detected and analyzed protein changes that behave in a coordinated way, providing information on functional category alterations (Zc) between the study groups. For further explanation, subgroups of categories were identified as most relevant in anaphylaxis. The selected categories were those with a number of proteins greater than 5 and less than 30, a log p value between -1.5 and 1.5, and those with -1.5 ≥ Zc mean ≥ 1.5.

## Results

### Proteome Identification of an *In Vitro* Anaphylaxis-Endothelial System

**Figure 3:**
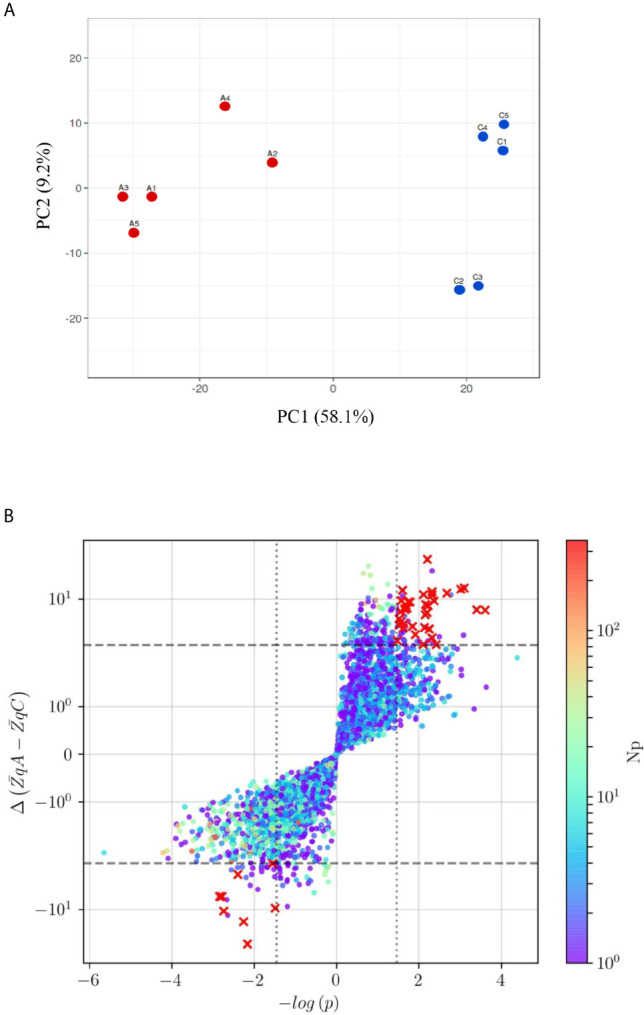
Principal Component analysis (PCA) and protein distribution of the EC-anaphylaxis- and control- sera/EC systems **(A)** PCA of the TMT10plex™ from the EC-anaphylaxis and EC-control set of proteins. Color coding: in red, the EC-anaphylaxis group (A1, A2, A3, A4, and A5) and in blue, the EC-control (C1, C2, C3, C4, C5). PC1, principal component 1; PC2, principal component 2. **(B)** The red crosses in the upper-right and lower-left quadrants indicate proteins presenting altered levels between EC-anaphylaxis and EC-control. The criteria applied were as follow: Np ≥ 3, color scale; |ΔZq| > 3, marked by the horizontal dashed line and Student’s t test with logarithmic transformation (|-log (p)> 1.5), delimited by the vertical dotted line. Y-axis and color scale are presented in logarithmic scale. Np, number of peptides; ΔZq, mean difference of abundance.

The direct impact of sera from anaphylactic and control subjects on primary ECs was evaluated through isobaric labeling and un-targeted mass spectrometry. A total of 7,707 proteins were identified (**Supplemental Table 1**). Among them, 1,069 were found to have a statistically significant difference between EC-anaphylaxis and EC-control and can be considered as an endothelial proteome in response to DIA. The PCA revealed grouping of biological replicates and separation of anaphylaxis and control groups by PC1 (58.1%) (**Figure 3A**). After applying the defined selection criteria, a scatter plot of 47 proteins exhibited the highest significant differences between the EC-anaphylaxis and EC-control groups (red crosses in **Figure 3B**).

### Anaphylactic Endothelial Subproteoma

**Figure 4:**
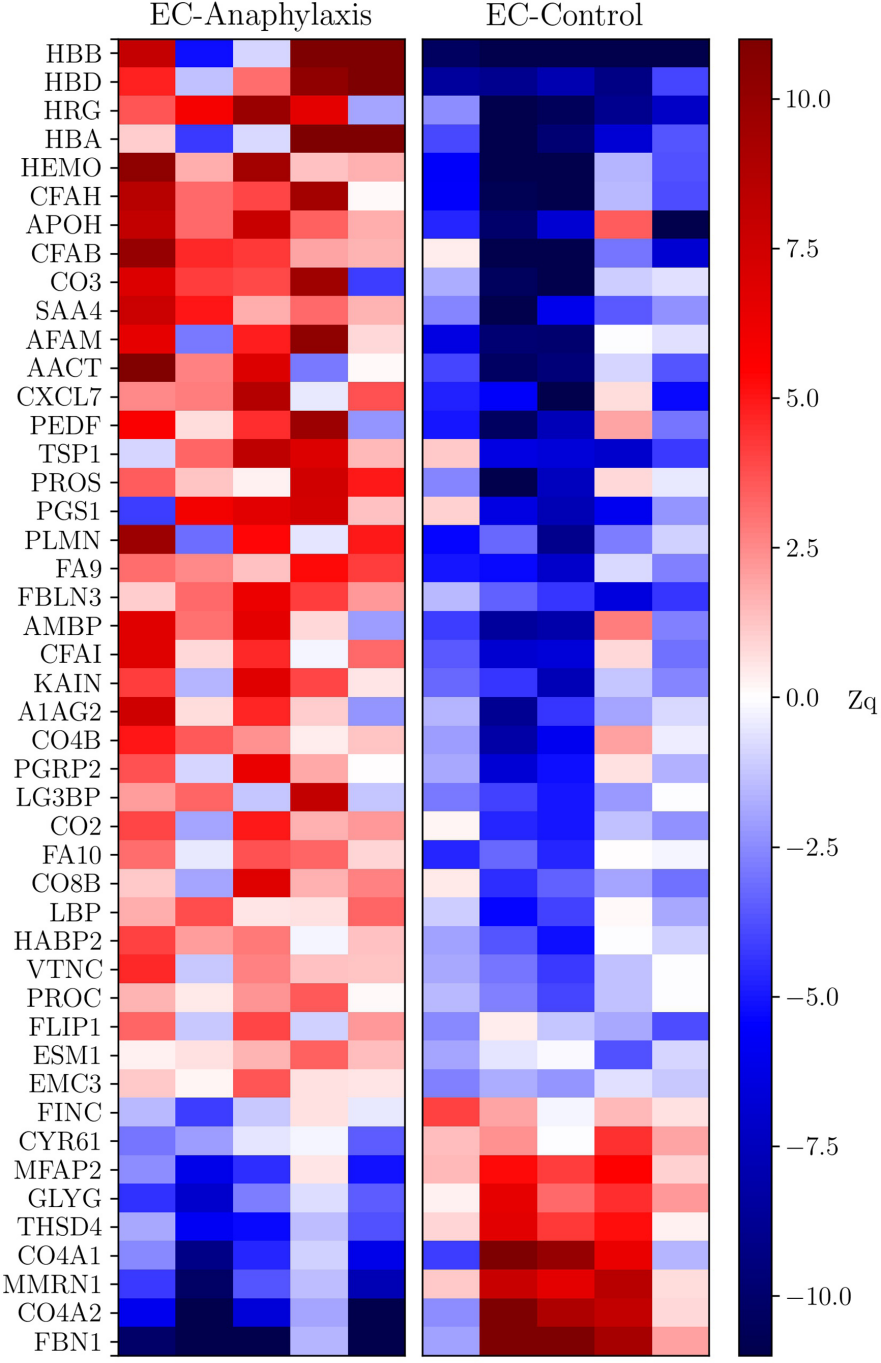
Heatmap of the data set of proteins selected in EC-anaphylaxis and EC-control systems. The y-axis corresponds to the statistically significant proteins and the x-axis to the mean abundance (ΔZq) in the samples from the EC-anaphylaxis group on the left and from the EC-control group on the right. According to the color scale on the right, increased proteins are indicated in red and decreased proteins in blue.

The profile of these 47 highly altered proteins is represented in a heatmap, showing 38 of them as increased and 9 as decreased in the EC-anaphylaxis subproteome (**Figure 4**). The β and δ hemoglobin subunits presented the highest positive variation rate (ΔZq = 28.96 and ΔZq = 13.39, respectively). Within the group of 9 decreased proteins, 7 of them are involved in cellular structural support; among them, fibrillin-1 (FBN-1), the α-2/1 (IV) collagen chains (CO4A2 and 1), and multimerin-1 (MMRN1) exhibited the highest down-regulation in EC-anaphylaxis. Furthermore, other proteins related to the cellular adhesive scaffold showed decreased levels in EC-anaphylaxis, such as fibronectin (FINC), CYR61 protein, microfibril-associated protein 2, and protein 4, containing the thrombospondin domain 1. As for the others, the quantitative proteomic analysis performed here indicated that proteins belonging to the coagulation and complement systems are increased in EC-anaphylaxis: coagulation factors IX and X, vitamin K-dependent protein C and S (PROC and PROS), β-2-glycoprotein 1 (APOH), thrombospondin-1 (TSP1), histidine-rich glycoprotein (HRG), plasminogen (PLMN), platelet basic protein (CXCL7), hyaluronan-binding protein 2 (HABP2), the complement components (C2, C3, C4B, C8 and factor B), and regulators (vitronectin and factors H and I) (**Table 2**).

**Table 2:** Dataset and abundance values of the proteins identified in the anaphylactic endothelial subproteome.

AccessionNumbers		Gen	Protein	Description	Np	Zq A	Zq C	Δ Zq	-log (p)
P68871	*HBB*		HBB	Hemoglobin subunit beta	10	12.08	-16.89	28.96	2.20
P02042	*HBD*		HBD	Hemoglobin subunit delta	6	5.70	-7.69	13.39	3.10
P04196	*HRG*		HRG	Histidine-rich glycoprotein	13	4.77	-8.30	13.07	3.02
P69905	*HBA1*		HBA	Hemoglobin subunit alpha	8	5.66	-7.06	12.71	1.60
P02790	*HPX*		HEMO	Hemopexin	22	4.89	-7.35	12.24	2.32
P08603	*CFH*		CFAH	Complement factor H	49	5.06	-6.70	11.76	2.68
P02749	*APOH*		APOH	Beta-2-glycoprotein 1	10	4.84	-6.41	11.25	2.11
P00751	*CFB*		CFAB	Complement factor B	23	4.47	-6.72	11.18	2.32
P01024	*C3*		CO3	Complement C3	101	4.09	-5.47	9.57	1.57
P35542	*SAA4*		SAA4	Serum amyloid A-4 protein	5	3.86	-5.67	9.53	2.37
P43652	*AFM*		AFAM	Afamin	22	3.91	-5.34	9.25	1.74
P01011	*SERPINA3*		AACT	Alpha-1-antichymotrypsin	16	3.53	-5.68	9.21	1.78
P02775	*PPBP*		CXCL7	Platelet basic protein	5	3.42	-5.20	8.63	2.16
P36955	*SERPINF1*		PEDF	Pigment epithelium-derived factor	10	3.64	-4.81	8.44	1.69
P07996	*THBS1*		TSP1	Thrombospondin-1	83	3.82	-4.62	8.44	2.22
P07225	*PROS1*		PROS	Vitamin K-dependent protein S	13	3.49	-4.27	7.76	1.71
P21810	*BGN*		PGS1	Biglycan	15	3.44	-4.25	7.69	1.66
P00747	*PLG*		PLMN	Plasminogen	32	3.25	-4.28	7.53	1.66
P00740	*F9*		FA9	Coagulation factor IX	6	3.31	-4.20	7.51	3.40
Q12805	*EFEMP1*		FBLN3	EGF-containing fibulin-like extracellular matrix protein 1	28	3.38	-4.02	7.40	3.61
P02760	*AMBP*		AMBP	Protein AMBP	13	3.02	-4.13	7.14	1.56
P05156	*CFI*		CFAI	Complement factor I	11	3.04	-3.88	6.92	2.19
P29622	*SERPINA4*		KAIN	Kallistatin	7	2.77	-3.82	6.59	2.15
P19652	*ORM2*		A1AG2	Alpha-1-acid glycoprotein	6	2.34	-3.51	5.85	1.52
P0C0L5	*C4B*		CO4B	Complement C4-B	4	2.52	-2.91	5.44	1.56
Q96PD5	*PGLYRP2*		PGRP2	N-acetylmuramoyl-L-alanine amidase	7	2.21	-2.98	5.18	1.64
Q08380	*LGALS3BP*		LG3BP	Galectin-3-binding protein	7	2.18	-2.83	5.00	1.51
P06681	*C2*		CO2	Complement C2	8	2.18	-2.63	4.81	1.84
P00742	*F10*		FA10	Coagulation factor X	4	2.11	-2.57	4.68	2.14
P07358	*C8B*		CO8B	Complement component C8 beta chain	6	2.09	-2.48	4.56	1.62
P18428	*LBP*		LBP	Lipopolysaccharide-binding protein	3	2.03	-2.45	4.48	2.25
Q14520	*HABP2*		HABP2	Hyaluronan-binding protein 2	7	2.03	-2.40	4.43	2.25
P04004	*VTN*		VTNC	Vitronectin	20	1.76	-2.07	3.84	1.91
P04070	*PROC*		PROC	Vitamin K-dependent protein C	3	1.61	-1.91	3.52	2.32
Q7Z7B0	*FILIP1*		FLIP1	Filamin-A-interacting protein 1	4	1.45	-1.83	3.28	*1.47*
Q9NQ30	*ESM1*		ESM1	Endothelial cell-specific molecule 1	6	1.48	-1.47	*2.95*	2.10
Q9P0I2	*EMC3*		EMC3	ER membrane protein complex subunit 3	5	1.24	-1.70	*2.94*	2.42
P02751	*FN1*		FINC	Fibronectin	109	-1.32	1.59	*-2.91*	-1.55
O00622	*CYR61*		CYR61	Protein CYR61	23	-1.85	2.03	-3.89	-2.40
P55001	*MFAP2*		MFAP2	Microfibrillar-associated protein 2	3	-3.52	3.50	-7.01	-2.78
P46976	*GYG1*		GLYG	Glycogenin-1	4	-3.69	3.35	-7.04	-2.85
Q6ZMP0	*THSD4*		THSD4	Thrombospondin type-1 domain-containing protein 4	9	-3.63	3.48	-7.11	-2.83
P02462	*COL4A1*		CO4A1	Collagen alpha-1(IV) chain	8	-4.70	4.89	-9.60	*-1.49*
Q13201	*MMRN1*		MMRN1	Multimerin-1	62	-5.47	4.95	-10.42	-2.74
P08572	*COL4A2*		CO4A2	Collagen alpha-2(IV) chain	19	-7.75	6.08	-13.83	-2.26
P35555	*FBN1*		FBN1	Fibrillin-1	101	-15.62	9.52	-25.14	-2.16

The color scale represents the increase (red) and decrease (blue) of each condition. Np, number of peptides; A, EC-anaphylaxis group; C, EC-control group; Zq, average of abundance values; ΔZq mean difference of the values. Established criterion Np ≥ 3, |ΔZq| ≥ 3, |-log (p) | ≥ 1.5.

**Figure 5:**
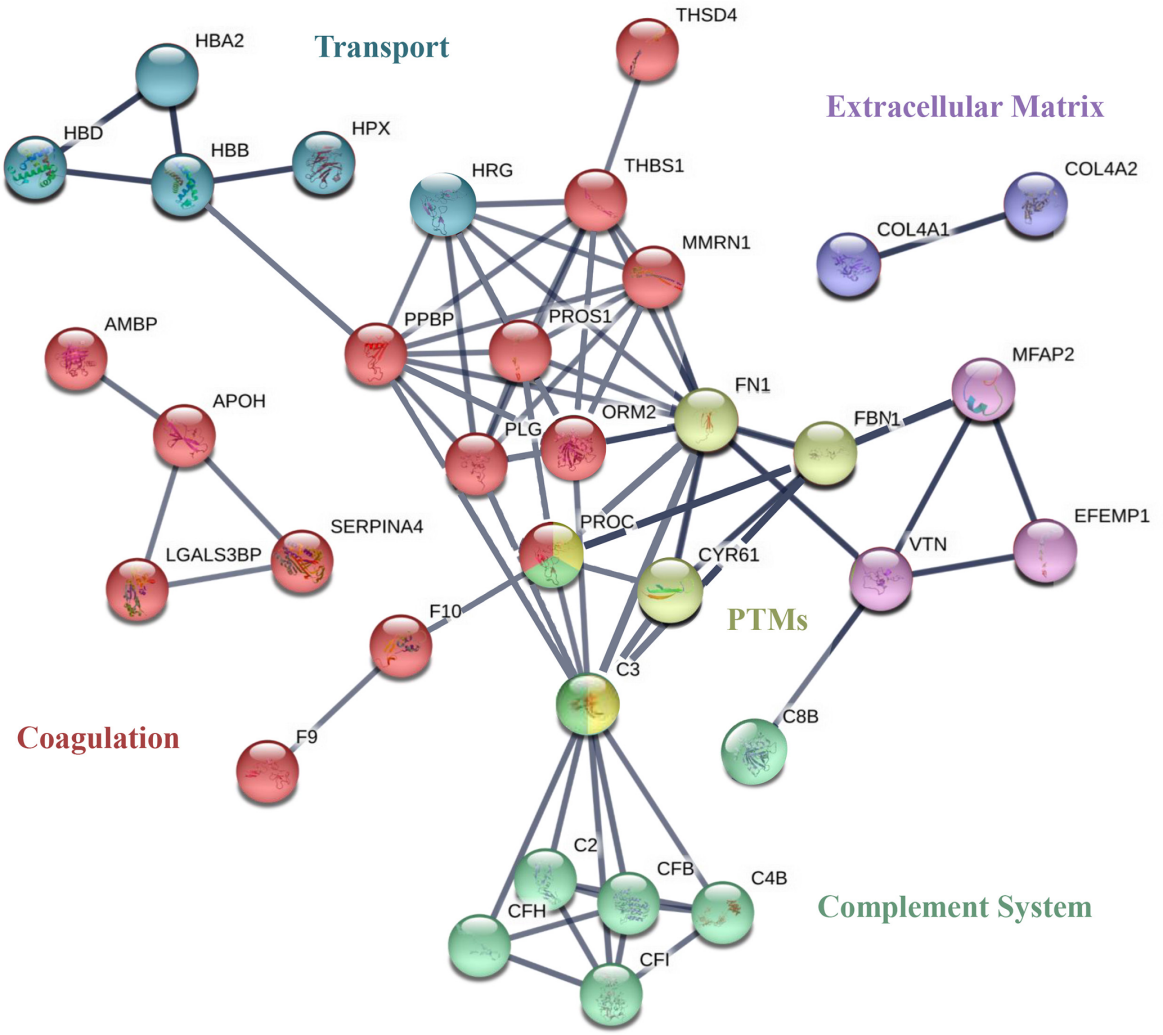
STRING of the most relevant proteins identified in EC-anaphylaxis and EC-control systems. Network analysis of the selected proteins found to be differential. The nodes of the networks represent individual proteins and the lines depict associations between proteins upon functional enrichment analysis.

To visualize the functional associations between these 47 individual proteins, a functional enrichment analysis was carried out by using the STRING v11 software tool (**Figure 5**). Blood coagulation (red nodes) and the complement cascade (green nodes) were identified as the biological processes with lowest FDR (7 · 10^-19^) and the higher number of identified proteins involved. Three sub-processes are included in the coagulation network: platelet degranulation (FDR = 5 · 10^-13^), the formation of fibrin clots formation (FDR = 2 · 10^-5^), and coagulation regulation (FDR = 5 · 10^-8^). The complement cascade node was composed of the following subsequent subprocesses: complement activation and its regulation (FDR of 4 · 10^-12^ and 10^-10^ respectively) and the regulation of the inflammatory response (FDR = 4 · 10^-9^). Moreover, 3 other biological processes were identified as contributing to anaphylaxis deregulation. Oxygen transport and heme group (blue nodes, FDR = 0.0001 and FDR = 0.0027, respectively), regulation of the biological activity by protein post-translational modification (PTMs; yellow nodes, FDR of 0.0113) and, at the cellular level, the regulation of the extracellular matrix (ECM) stood out with an FDR of 10^-9^ (purple and violet nodes).

### The Complement Is the Primary Altered Biological System

**Figure 6:**
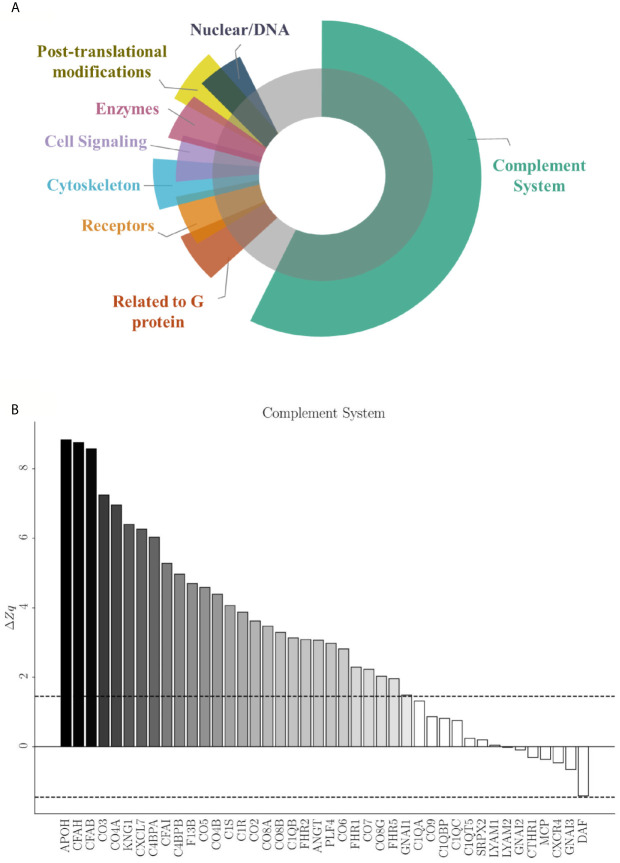
Specific protein variation contributing to complement system alteration in EC-anaphylaxis. **(A)** Main altered functional categories in EC-anaphylaxis. **(B)** The graphic represents the list of proteins identified in relation with the complement system and their specific ΔZq.

The coordinated response of the total endothelial proteome by means of an SBA resulted in 5,513 identified functional categories (**Supplemental Table 2**). Among them, 57 presented significant differences after applying the selection criteria. **Figure 6A** shows the categories grouped and their corresponding subcategories (**Table 3**) found to be statistically altered between EC-anaphylaxis and EC-control.

**Table 3:** Main altered functional categories in EC-anaphylaxis compared to EC-control system.

Category	Subcategory	Zc A	Zc C	Δ Zc	- log (p)
**Complement** **System**	Complement component	12.72	-14.33	27.05	1.82
	Complement component 5	7.83	-11.17	19.00	1.67
	Complement component-related SUSHI domain-containing	10.86	-10.03	20.89	1.87
**Related to G Protein**	GNAS complex locus	0.98	-1.05	2.04	1.7
	Rap1 complex	-1.17	1.01	-2.18	-2.17
**Receptors**	5HT2 type receptor mediated signaling pathway	0.91	-1.01	1.92	2.16
	Histamine H2 receptor mediated signaling pathway	0.69	-0.87	1.56	1.78
	Thyrotropin-releasing hormone receptor signaling pathway	1.00	-0.93	1.94	2.3
	Tlr 1/2	-0.78	1.12	-1.90	-1.78
**Cytoskeleton**	Kinesin transport	-0.81	0.93	-1.74	-2.46
	TCR actin (T cell receptor)	-0.93	1.10	-2.03	-2.38
	DRG1 complex	-1.01	1.36	-2.38	-1.95
	Actin binding motor protein	-1.47	1.23	-2.70	-2.55
	Myosin	-1.46	1.29	-2.75	-3.36
**Cell Signaling**	JAK-STAT cascade	-0.85	0.65	-1.50	-2.08
	STAT1 complex	-0.81	0.99	-1.80	-1.54
	Cell proliferation	-0.82	1.20	-2.02	-2.47
	Cytotoxicity of leukocytes	-0.85	1.21	-2.06	-2.57
	Interferon	-1.36	1.33	-2.69	-1.72
	Interferon-mediated immunity	-1.32	1.50	-2.82	-1.77
**Enzymes**	Glycosidase	1.02	-1.19	2.21	2.06
	Kinase inhibitor	0.77	-1.02	1.79	2.24
	Phospholipases	0.37	-1.26	1.63	1.95
	Lymphocyte-specific protein tyrosine kinase	0.77	-0.89	1.66	1.82
	tRNA ligase	-0.85	0.94	-1.79	-2.37
	Multisynthetase complex	-0.74	0.90	-1.64	-2.72
	Kinase maturation complex 1	-1.04	1.37	-2.41	-1.77
**PTMs**	Ubiquitin-conjugatin enzyme E2	1.05	-1.10	2.15	2.43
	Sumo1 complex	-0.79	0.82	-1.61	-1.84
	E3 ubiquitin ligase	-0.99	0.96	-1.95	-3.54
**Nuclear/DNA**	Set1A complex	0.84	-1.06	1.90	2.17
	CSA-POLIIa complex	-0.76	0.84	-1.60	-1.75
	TERF2-RAP1 complex	-0.94	0.71	-1.65	-1.54
	H2AX complex I	-0.67	1.03	-1.70	-1.61
	U5 snRNP complex	-0.81	0.91	-1.71	-1.75
	DDB2 complex	-0.79	0.95	-1.74	-1.64
	Other nucleic acid binding	-1.05	0.83	-1.88	-2.13
	TRBP containing complex	-0.99	1.00	-2.00	-1.98
	XRCC5 complex	-0.97	1.19	-2.16	-1.66
	Cell cycle: G2-M DNA damage checkpoint regulation	-0.94	1.34	-2.28	-1.91
	SNW1 complex	-0.79	1.50	-2.29	-1.82
	NCOA6-DNA-PK-Ku-PARP1 complex	-1.22	1.17	-2.40	-1.68
	DHX9-ADAR-vigilin-DNA-PK-Ku antigen complex	-1.21	1.31	-2.52	-1.92
	DNA repair	-1.32	1.45	-2.77	-2.39
	TLE1 corepressor complex	-1.58	2.05	-3.63	-2.85
**Other**	Other extracelular matrix	2.13	-4.19	6.32	1.78
	Affects N-acetylglucosamine metabolism	0.98	-1.12	2.10	2.19
	Heterotrimeric G-protein signaling pathway-rod outer segment phototransduction	0.51	-1.14	1.65	2.9
	nNOS signaling in skeletal muscle cells	-0.75	0.76	-1.51	-2.66
	Renal glomerulus panel	-0.79	0.90	-1.70	-2.1
	Increase glucosa metabolism	-0.82	0.94	-1.76	-1.83
	Hearing	-0.79	0.97	-1.77	-1.63
	Phagocytosis	-0.88	1.01	-1.89	-2.08
	*De novo* purine biosynthesis	-0.91	1.06	-1.97	-1.63
	Signal recognition particle	-1.04	1.00	-2.04	-1.58
	Hematopoiesis	-0.85	1.45	-2.30	-1.51
	Asthma	-1.37	0.99	-2.37	-2.21

The color represents the values of abundance increase (red) or decrease (blue) grouped in functional categories (Zc) using as selection criteria (|ΔZc|> 1.5) and Student’s t test with logarithmic transformation (|-log (p) | ≥ 1.5). Zc A, average of the Zc values in the EC-anaphylaxis group; Zc C, average of the Zc values in EC-control group.

Those 57 functional subcategories included a total of 785 proteins presenting a coordinated response, of which 407 were unique and 151 were common to several categories. In accordance with the results based on individual high-altered proteins of functional networks, SBA confirmed that the complement system is the main altered category in ECs incubated with sera from anaphylactic patients (**Figure 6B**). The number of proteins belonging to the complement system are part of: both the activation of the classical and alternative pathway, the terminal components complex (TCC; C5, C6, C7, C8 and C9), as well as regulators of the complement system. Specifically, APOH and DAF (decay-accelerating factor are respectively the proteins with the highest and lowest rates of change inside this set of proteins. Surprisingly, EC-anaphylaxis showed a moderated modulation of other categories related with G proteins, receptors, cell signaling, cytoskeleton, and enzymes, among others. The modulation of this set of proteins and their rates of change were represented individually for each category (**Supplemental Figure 1**). Therefore, relevant differences were seen in rate of changes between the complement category that present the major (ΔZc = 27.05) against the amount of other of categories presenting around (|ΔZc| ~ 2).

## Discussion

It is extensively described the relevance of the endothelium in vascular permeability associated to anaphylaxis is widely known. However, few studies focus in the participation of ECs as contributors of the vascular homeostasis and even less have used an untargeted approach covering ECs proteome. This work has identified protein patterns of an endothelial cell culture in response to sera collected at the acute moment of the anaphylactic reaction.

Most of the molecular mechanisms underlying vascular permeability and anaphylaxis has been identified in experimental models (41–43). We have previously demonstrated that two different endothelial molecules (Regulator of calcineurin 1 and Fibroblast growth factor–inducible molecule 14) participate in the regulation of the endothelial barrier function related with anaphylaxis (44, 45). Furthermore, the increased leakage has shown correlation with a marked decrease in blood pressure during anaphylaxis (46, 47). In the SBA performed here, a related set to cytoskeletal and G proteins have been identified as significantly modulated in the EC-anaphylaxis system together with cell signaling addressed by JAK-STAT cascade in line with previous observations showing STAT3 inhibition and vascular permeability reduction (48). However, the leakage and NO release are expected to occur very rapidly. Moreover, they associated to the fast action of well-known mediators of anaphylaxis, as histamine or PAF. As we have studied here an *in vitro* cellular system incubated with anaphylactic serums for a longer time, it is undoubted that other relevant events also stand out.

The individual protein analysis of our system shows that hemoglobin subunits and structural support proteins are those presenting the main alterations in response to anaphylaxis. It is known that free hemoglobin is able to cross the endothelial barrier and be internalized by ECs by endocytosis or transcytosis mechanisms (49). Here, we confirm an abundant increase in the β, δ and α subunits of these proteins in the EC-anaphylaxis system studied. The presence of these hemoglobin in ECs has a great impact in the pathophysiology of the vasculature in anaphylaxis being related with vasoconstriction, vascular permeability or extravasation (50–55). On the other hand, the ECM provides stable anchoring of factors and fibers which interact with cell surface receptors participating in proliferation, migration and survival processes of both ECs and VSMCs. Low levels of ECM structural proteins are related with disruption of the fibers interfering with cell adhesion, the blood-gas barrier and endothelial permeability. In the EC-anaphylaxis system, we have observed FBN-1, CO4A2/1, and MMRN1 as the most decreased proteins. These data show the deleterious impact of anaphylaxis in the ECM comprising its stability. Besides, we also identify reduced levels of FINC in EC-anaphylaxis pointed once again to an ECM destabilization. This process may favor an increase in fluid extravasation, since in anaphylactic reactions it is expected that vascular permeability occurs very rapidly.

**Figure 7:**
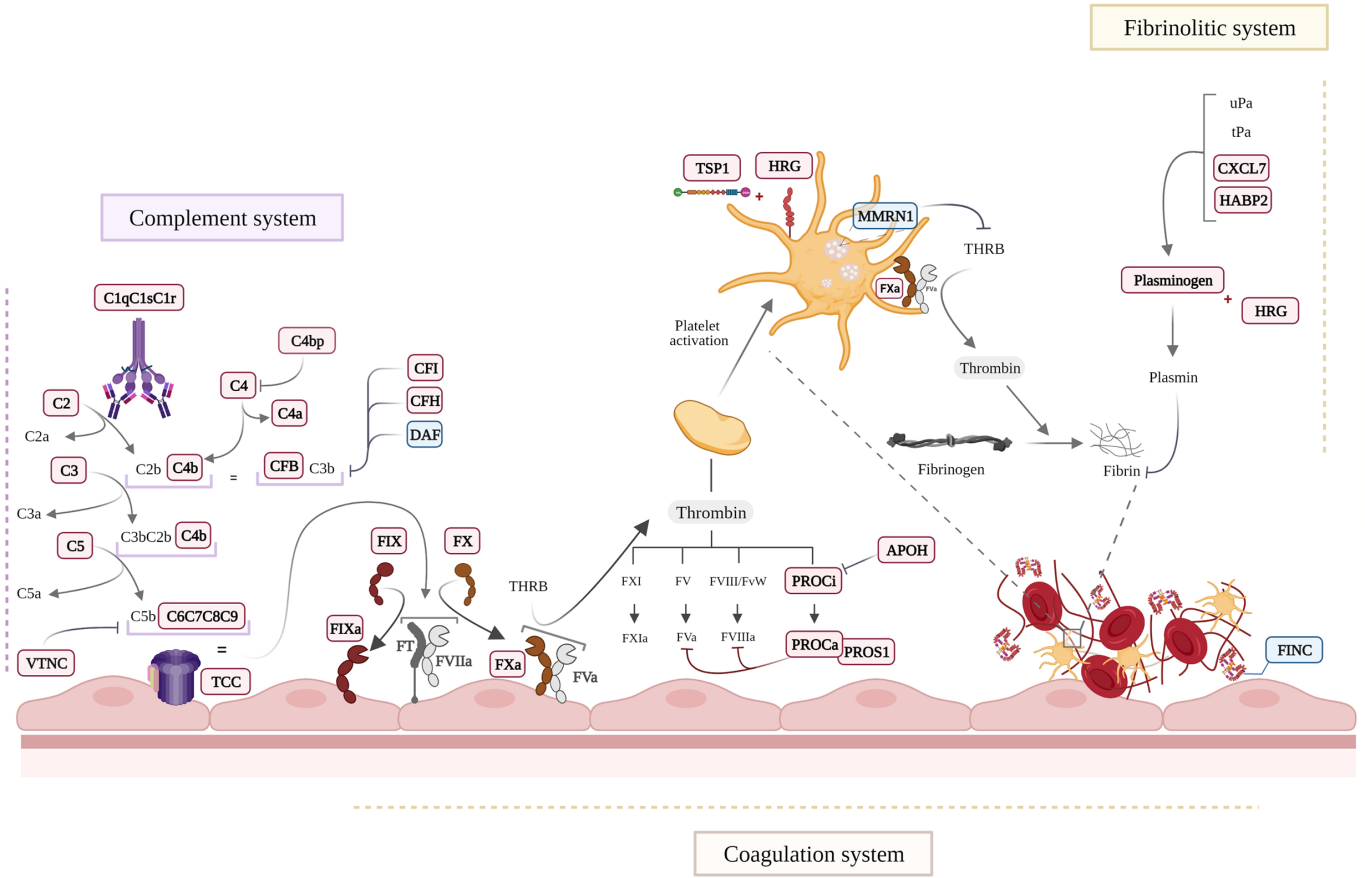
Schematic view of the main molecular processes affected in EC-anaphylaxis. Proteins appearing as in bold and red are increased and those in blue were decreased in the EC-anaphylaxis group.

The coagulation, fibrinolysis, contact and complement systems are activated simultaneously during systemic inflammation, severe tissue injury or acute trauma (25, 56). A significant number of coagulation and complement systems proteins has been identified in our studies, both as the main modulated individual proteins but also as main functional networks operating in EC-anaphylaxis (**Figure 7**). Specifically, we observed upregulated levels of the factors IX and X. However, low values ​​of coagulation factors in sera samples from anaphylactic patients has been reported before and can be related to what is happening at cellular level taking into account our observations (57–59). Other protein related with coagulation processes have been found increased in EC-anaphylaxis: PROC, PROS, APOH, Galectin-3 and TSP1. These protein increments in response to anaphylaxis suggest that the endothelium present a dual role not only a prothrombotic state in the anaphylactic reactions but also as an anticoagulant active surface. The activation of PLMN, the main molecule involved in fibrinolysis, has been previously correlated with hypotension during anaphylaxis (60–62). Accordingly, PLMN and two of its activator proteins were identified in EC-anaphylaxis: CXCL7 and HABP2. In addition, PLMN anchors to cell membranes by binding to HRG, which, in fact, is the third most upregulated protein identified in our panel.

Activation of the coagulation/fibrinolytic system usually takes place together with the activation of the complement system, which is considered a triggering mechanism of anaphylactic shock. Different agents or anaphylaxis triggers can be considered complement activators (63). High levels of C3a and C4a (for years referred to as anaphylatoxins) were nearly fatal for an anaphylactic patient despite the absence of changes in mediators, effector cells, or other systems (64). Importantly, the deficiency or inactivation of C3 or C4 prevents anaphylactic symptoms in mice (65, 66). Mechanistically, the release of these inflammatory molecules exerts profound effects on the cardiovascular system through their receptors present in mast cells, basophils and ECs (67, 68). In this context, it is relevant that mediators released by mast cells, such as tryptase, in turn generate complement amplification through an activation loop independent of the system’s own convertases (69). In general, C3a and C4a are considered mediators released during anaphylaxis that stimulate VSMC contraction, increase vascular permeability, cause pulmonary vasodilation and hyperresponsiveness of the airways, among other consequences (70–73). The quantitative proteomic analysis performed here indicates that proteins belonging to the complement system are increased in EC-anaphylaxis confirming the relevance of the endothelial niche in the activation of the complement system during anaphylaxis (**Figure 7**). Most of the complement proteins identified with high alteration belong to the classical and alternative activation pathways. However, when a coordinated response of the proteins identified is performed (SBA) the importance of TCC highlights being related with EC-anaphylaxis for the first time. Furthermore, the SBA study highlights relevant changes of TCC in EC-anaphylaxis for the first time. The involvement of the complement system in the development of anaphylaxis is correlated with the severity of the reactions, and those more severe are classified by including the involvement of the cardiovascular affectation in the reactions (1, 74). Therefore, ECs could also be participating in turn as an extrahepatic source of both complement components and their regulators (75). For instance, in response to cytokines such as Interleukin-1, Interleukin-6, Tumor necrosis factor-α or Interferon-γ (most of them are mediators of anaphylaxis) (76–78).

This study presents some limitations, being one of them that around 20% of the identified proteins are described as mainly localized in blood. The cell/serum system was abundantly washed to avoid any contamination. Besides, looking in detail, many of these proteins are glycoproteins involved in the binding between the glycocalyx and the cellular membranes. Contamination cannot be, thus, completely discarded but, in case of existence, has been highly minimized. Furthermore, the own nature of the *in vitro *system makes the extrapolation of our results difficult with “the real events of an anaphylactic reaction”. However, it gives us a robust picture of the *in vitro* microenvironment created between serum and the endothelial monolayer. Other aspect is related to the number of samples and the lack of a validation cohort, since our study fulfils the requirements of an omics analysis. As it is known, the heterogeneity of the anaphylactic reactions makes its study enormously difficult. The main criteria followed here to select samples have attended to clinical signs and symptoms of anaphylaxis determined by allergists (1, 79). We highlight the severity and high tryptase values to determine the main homogeneity between the anaphylactic sampling choose. Severity grading systems and tryptase cut-offs values open plenty of avenues in order to validate our data in a bigger cohort of patients. Undoubtedly, a great advance in our studies would involve the classification of samples by comparing groups or discriminating other factors (as different triggers, mechanisms IgE/non IgE related, sex of the subjects, etc.). Importantly, several proteins and networks identified have not been discussed. They do not require less attention and open the door to extend these findings in future studies.

An accurate and rapid diagnosis is necessary for the correct management of anaphylaxis. Therefore, our research provides a totally novel approach to anaphylaxis combining a proteomic and a systems biology analysis that has led to a large amount of data. After an extensive statistical and biological processing, we provide here results that improve the knowledge of the anaphylaxis underlying mechanisms at cellular level pointing to new molecules and pathways related to ECs as targets.

## Data Availability Statement

The original contributions presented in the study are included in the article/**Supplementary Material**. Further inquiries can be directed to the corresponding author.

## Ethics Statement

The studies involving human participants were reviewed and approved by IIS-FJD Clinical Research Ethics Committee PIC38/2016, PIC142/016 and PIC057-19. The patients/participants provided their written informed consent to participate in this study.

## Author Contributions

Conceptualization, VE. Methodology, AY-M, SF-B, TO, CP-V, DB, MG and JAL. Formal analysis, AY-M, JAL and MM-L. Investigation, AY-M, GA-L and VE. Resources, VE, JC-H and JJL. Writing—original draft preparation, AY-M. Writing—review and editing, VE, GA-L and MM-L. Visualization, AY-M. Supervision, VE. Project administration, VE. Funding acquisition, AY-M, SF-B, TO, CP-V, DB, MG, JJL, JAL, GA-L, JC-H, MM-L and VE. All authors contributed to the article and approved the submitted version.

## Funding

This work was supported by the Spanish Council Ministry of Science and Innovation (MCIN) (RyC-12880-2013), grants from the Instituto de Salud Carlos III (ISCIII) PI16/00888, PI16/01334 and PI18/00348 and supported by RETICS ARADyAL (RD16/0006/0013, RD 16/0006/0031 and RD16/0006/0033), co-supported by FEDER grants. This work was also supported by the SEAIC (19_A08), Alfonso X el Sabio University Foundation, PRB3 [IPT17/0019-ISCIII-SGEFI/ERDF] and Fundación SENEFRO/SEN. DB is supported by a Rio Hortega Research Contract. AY-M and MML are supported by Consejería de Educación, Juventud y Deporte de la Comunidad de Madrid y del Fondo Social Europeo (PEJ- 2019-PRE/BIO-16915, PEJ-2017-PRE/BMD-5007 and 2018-T2/ BMD-11561 respectively) and the work is also supported by CM_P2018/BAAA-4574. The CNIC is supported by the ISCIII, MCIN, Pro CNIC Foundation, and is a Severo Ochoa Center of Excellence (SEV-2015-0505).

## Conflict of Interest

The authors declare that the research was conducted in the absence of any commercial or financial relationships that could be construed as a potential conflict of interest.

## Abbreviations

APOH, β-2-glycoprotein 1; CO4A2/1, α-2/1 (IV) collagen chains; CXCL7, Platelet basic protein; DIA, Drug-induced anaphylaxis; EC-anaphylaxis, Group of cells stimulated with sera from anaphylactic patients; EC-control, Group of cells stimulated with sera from control subjects; ECM, Extracellular matrix; ECs, Endothelial Cells; FBN-1, Fibrillin-1; FDR, False Discovery Rate; FINC, Fibronectin; HABP2, Hyaluronan-binding protein 2; HRG, Histidine-rich glycoprotein; MMRN1, Multimerin-1; NO, Nitric oxide; Np, Number of peptides; PLMN, Plasminogen; PROC, Vitamin K-dependent protein C; PROS, Vitamin K-dependent protein S; PTM, Post-translational modification of proteins; SBA, System Biology Analysis; SBT, Systems Biology Triangle; ST, Serum Tryptase; TCC, Terminal components complex; TSP1, Thrombospondin-1; VSMCs, vascular smooth muscle cells; Zc, Functional category; Zq, Abundances of the protein.
