# Supplementary material for: Proteomic and Biological Analysis of an In Vitro Human Endothelial System in Response to Drug Anaphylaxis
Source: Front Immunol. 2021 Jun 25;12:692569. doi: 10.3389/fimmu.2021.692569 (PMC8269062; doi:10.3389/fimmu.2021.692569)
Supplement: Supplementary file 1 [file DataSheet_1.pdf]

Supplemental Figure 1A

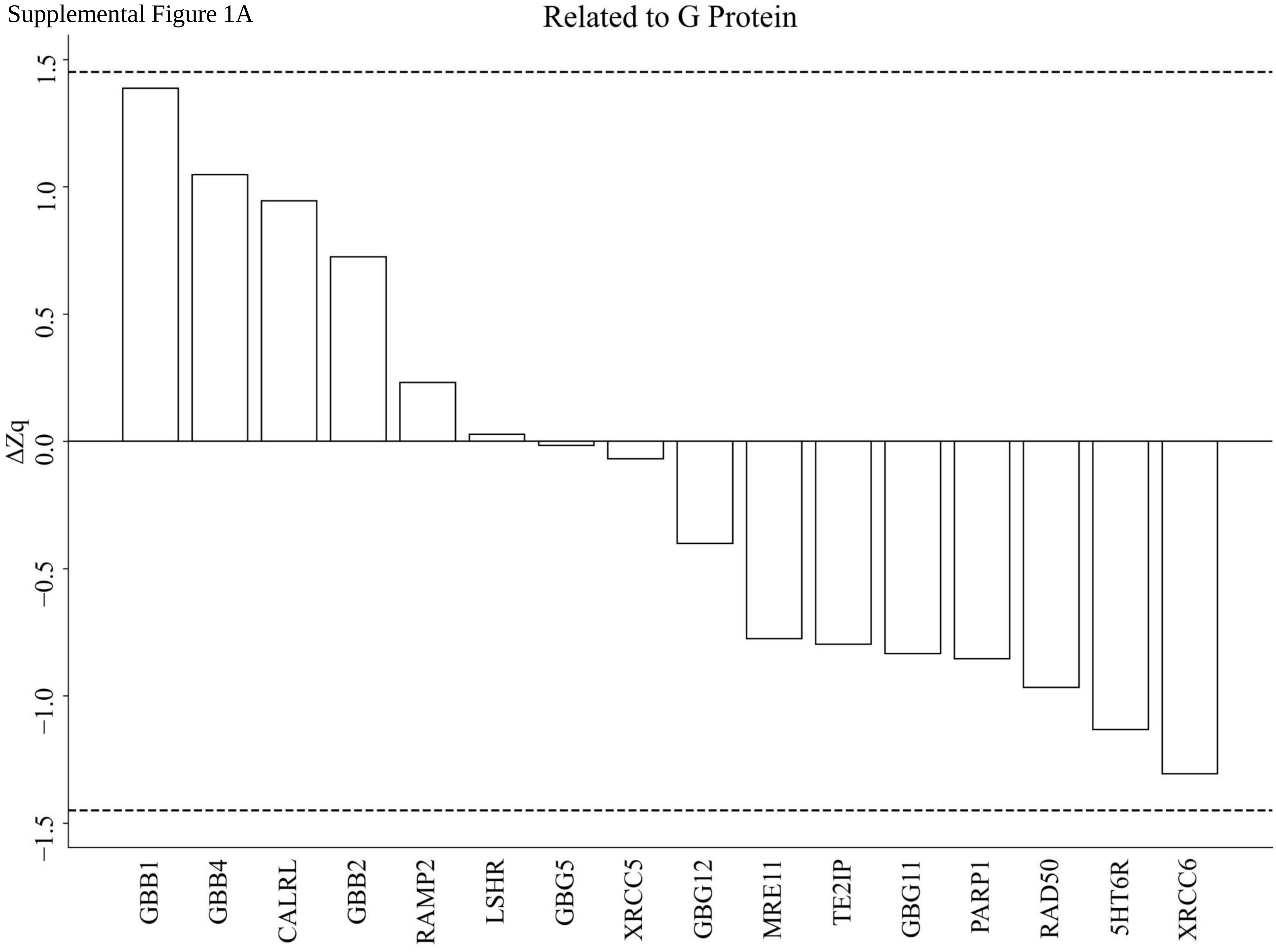

Supplemental Figure 1B

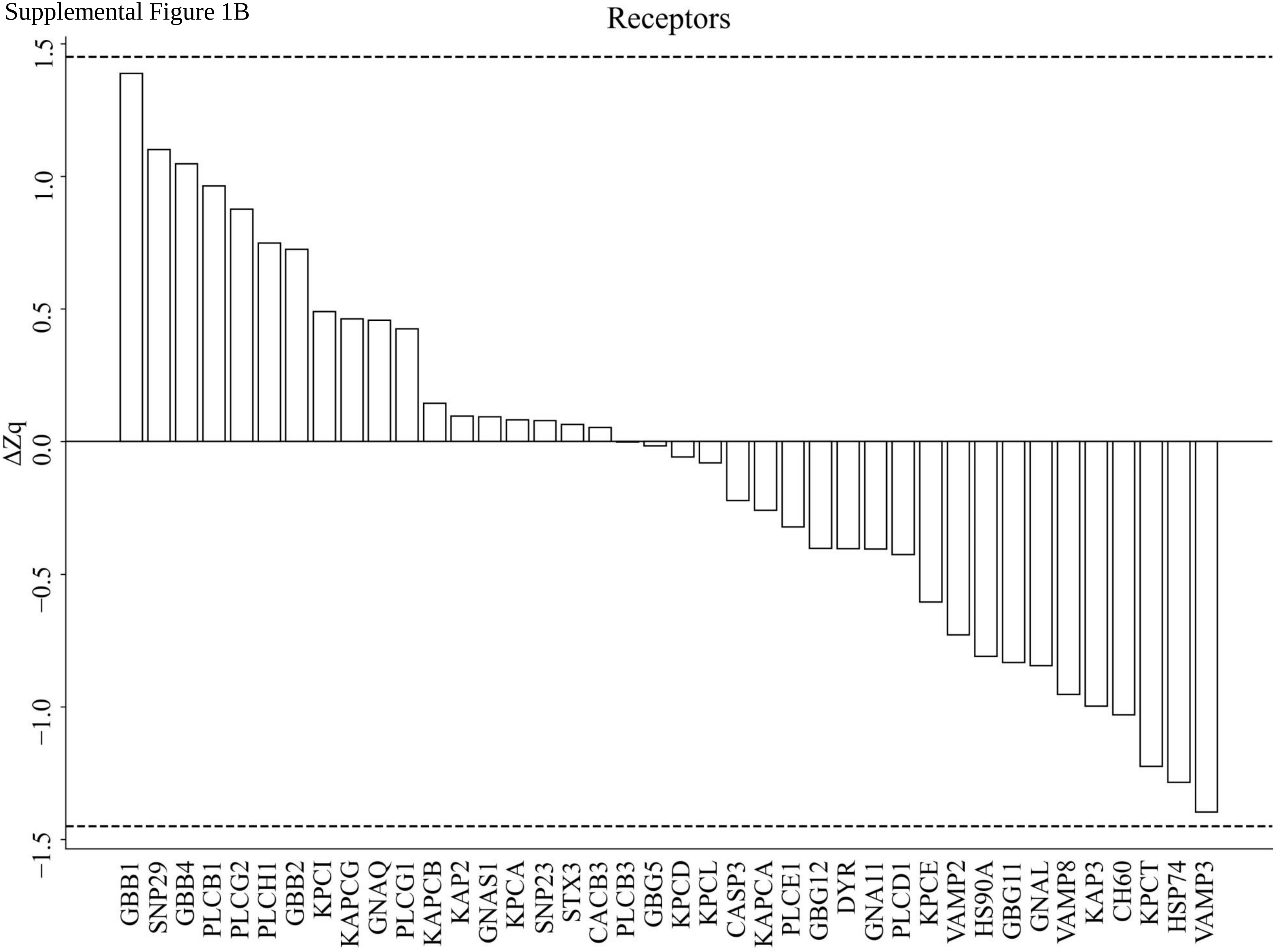

Supplemental Figure 1C

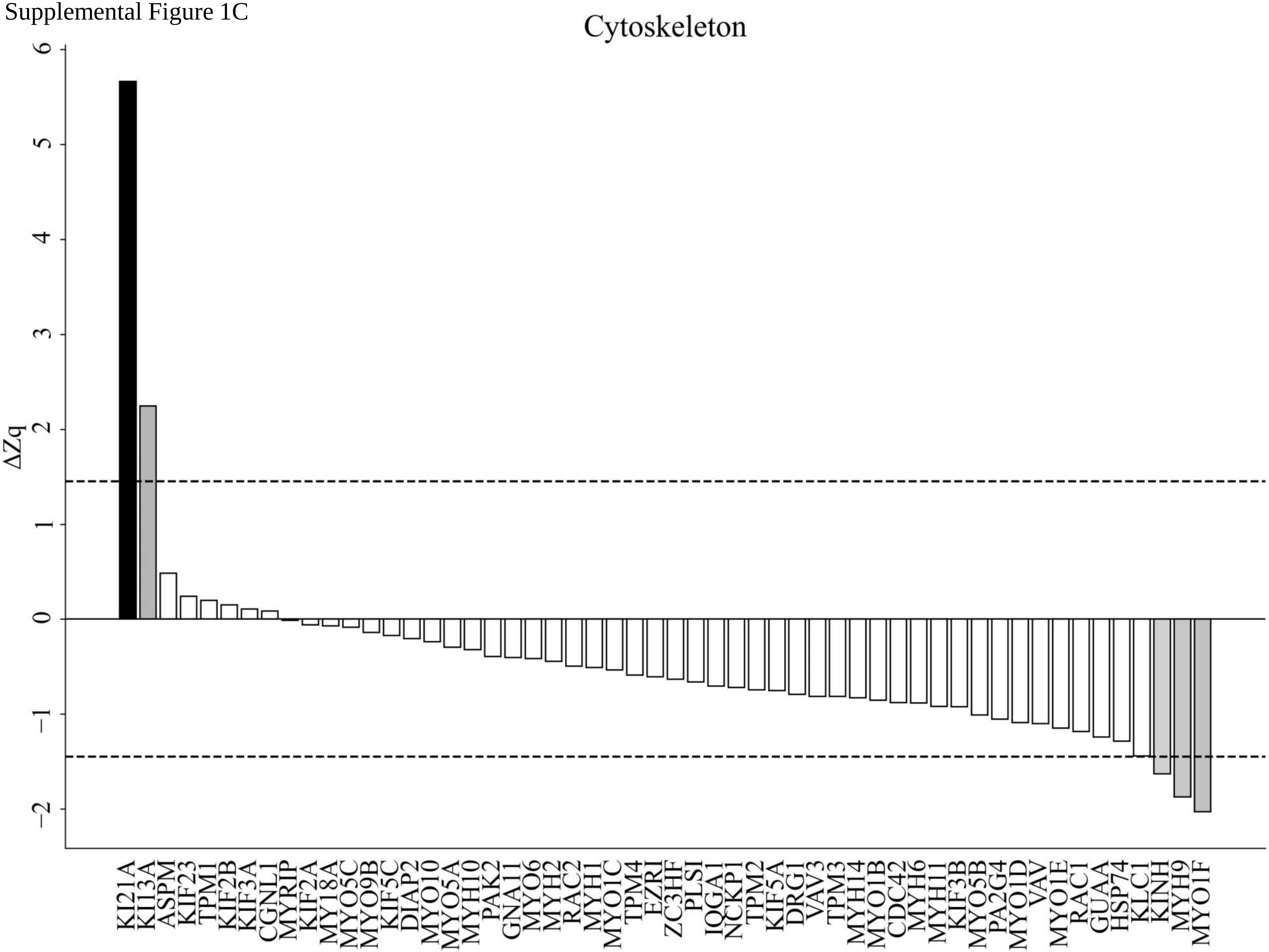

Supplemental Figure 1D

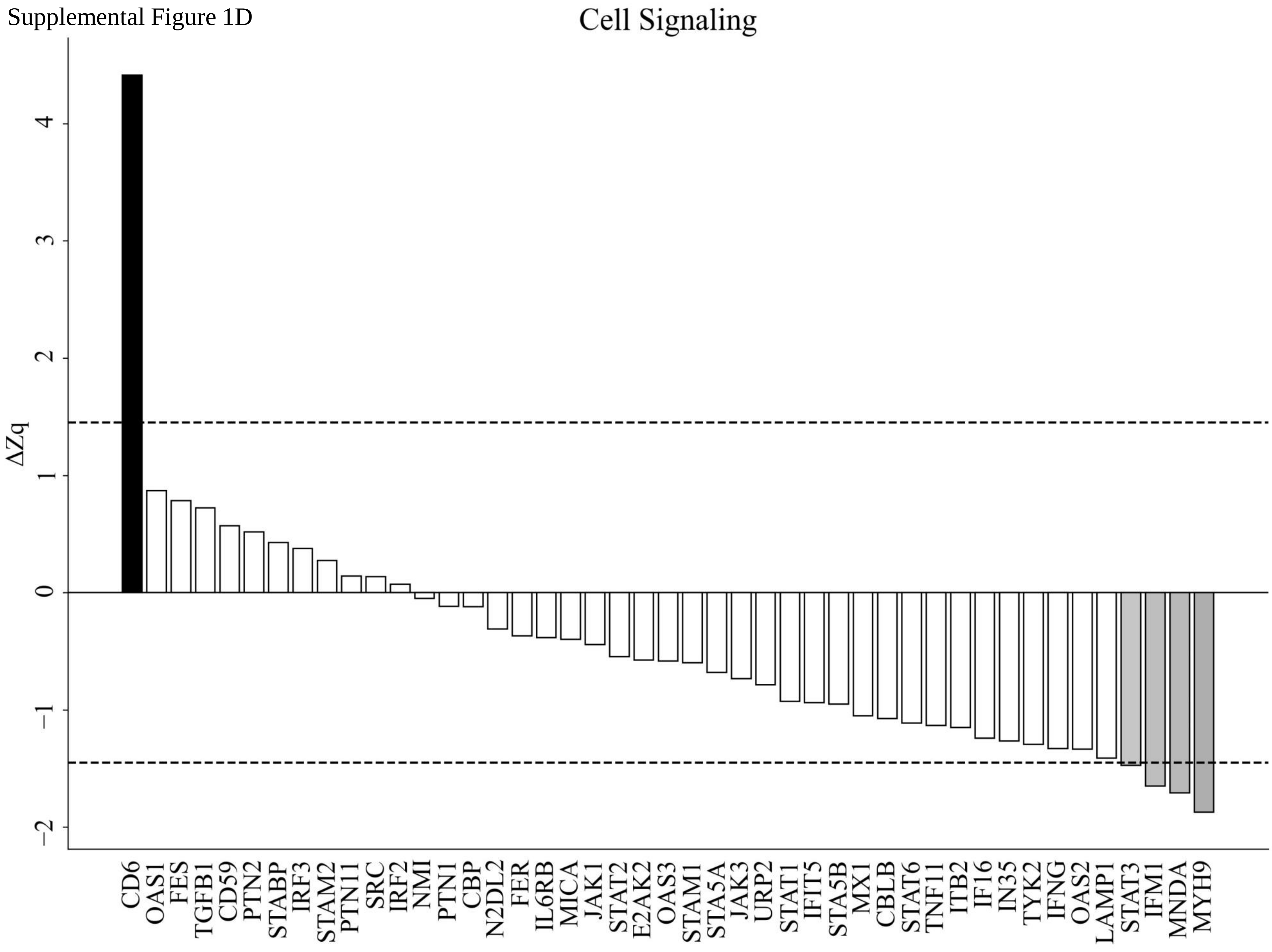

Supplemental Figure 1E

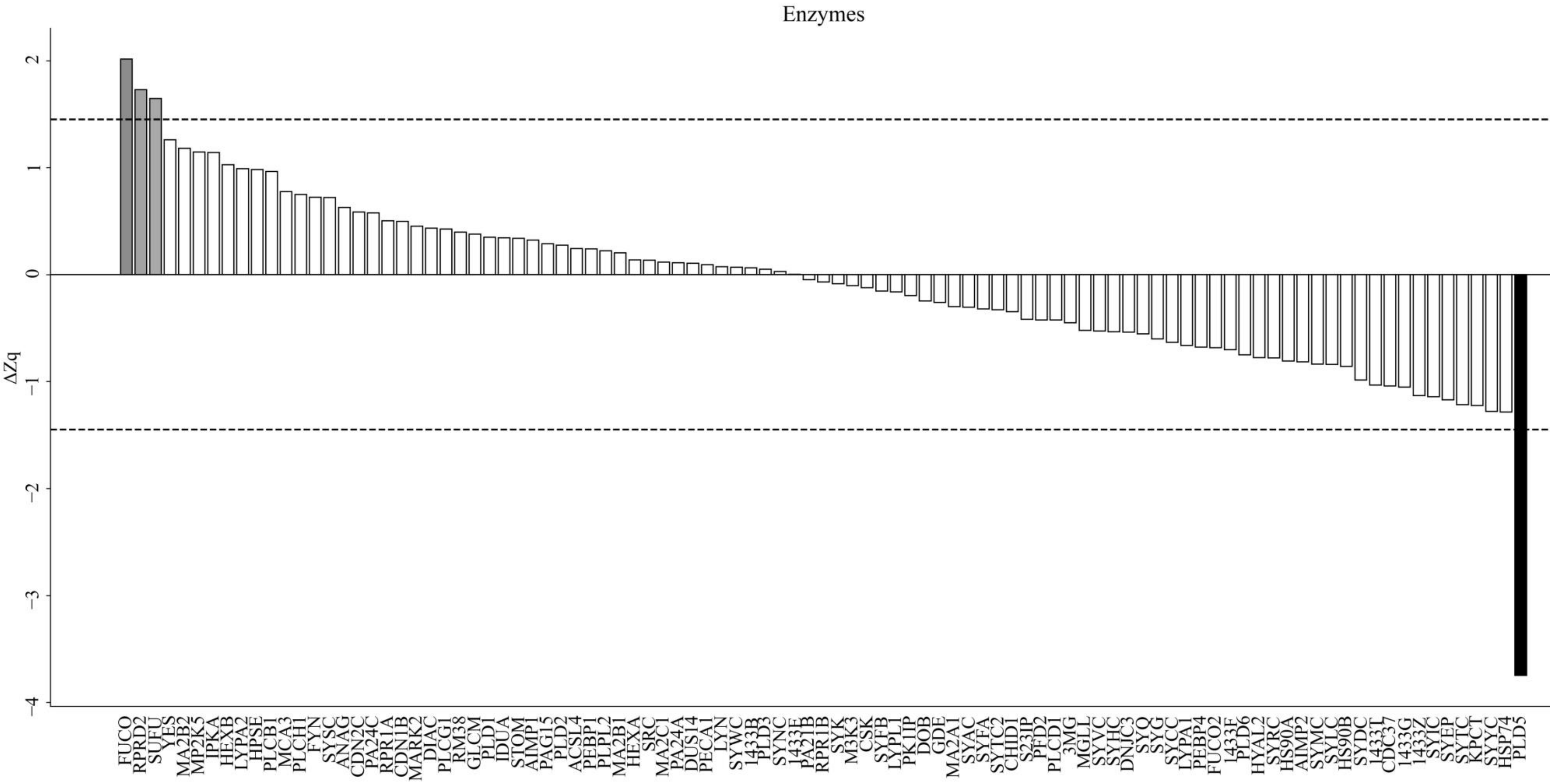

Supplemental Figure 1F

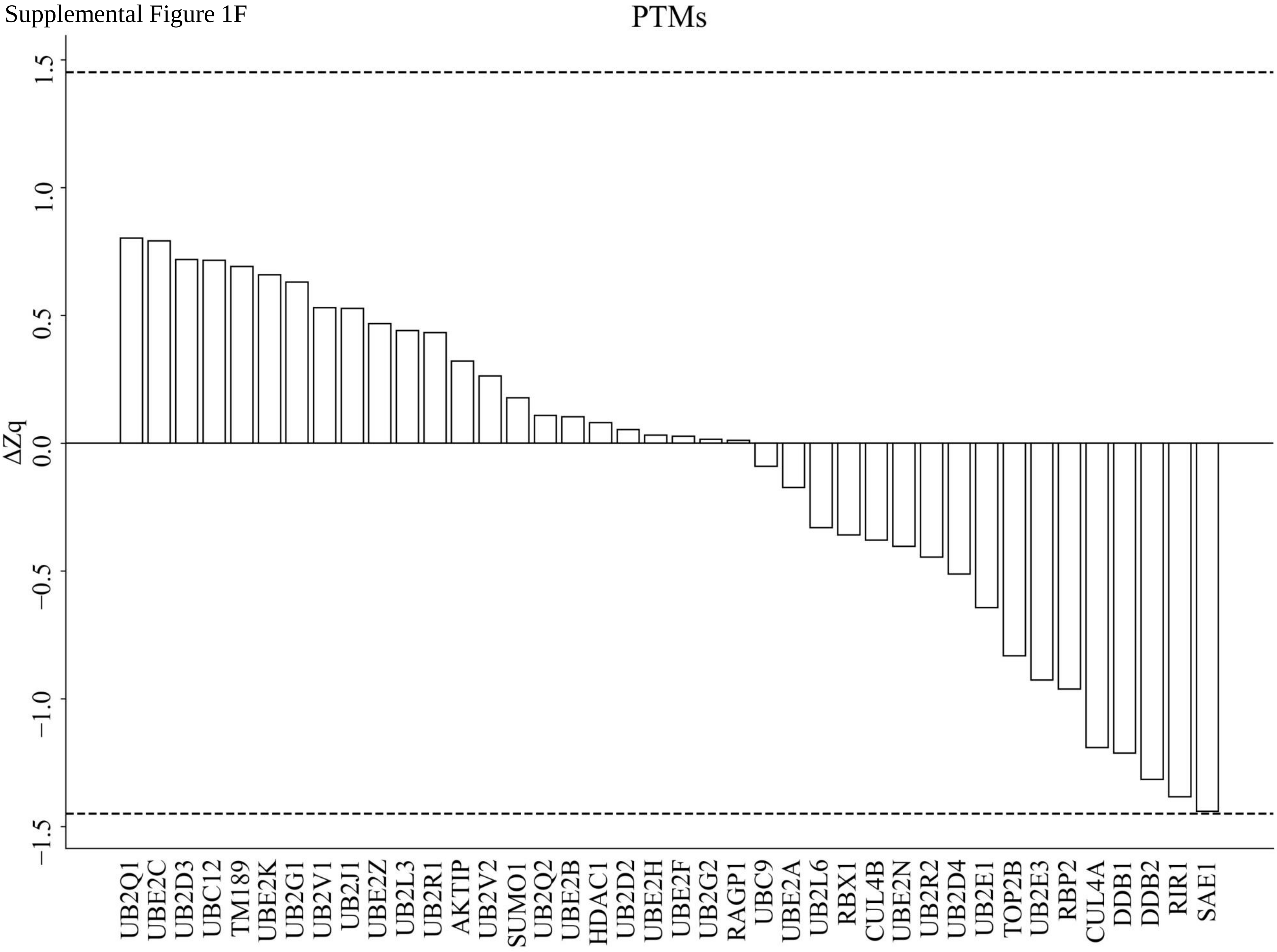

Supplemental Figure 1G

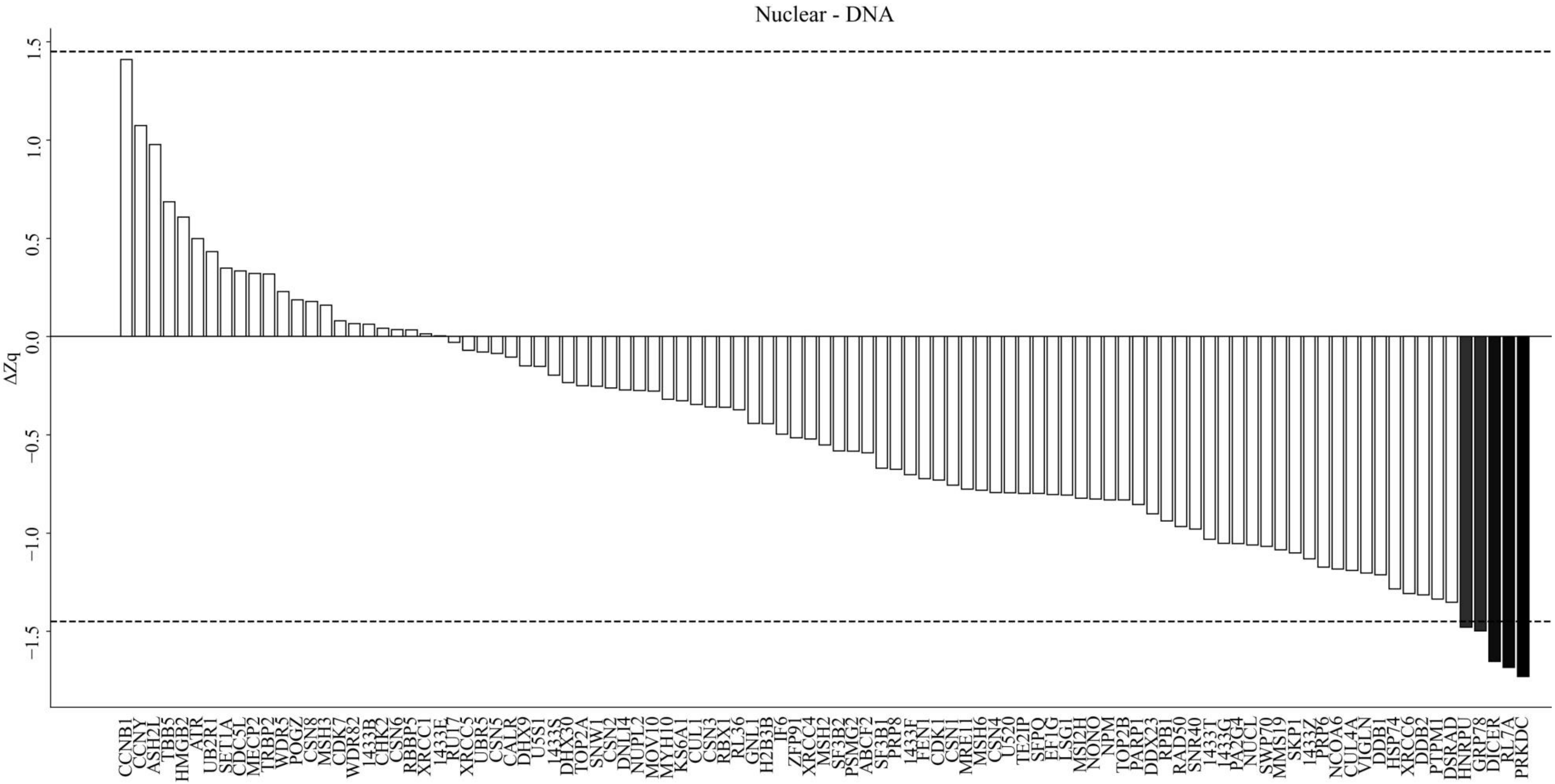

Supplemental Figure 1H

Others (1/2)

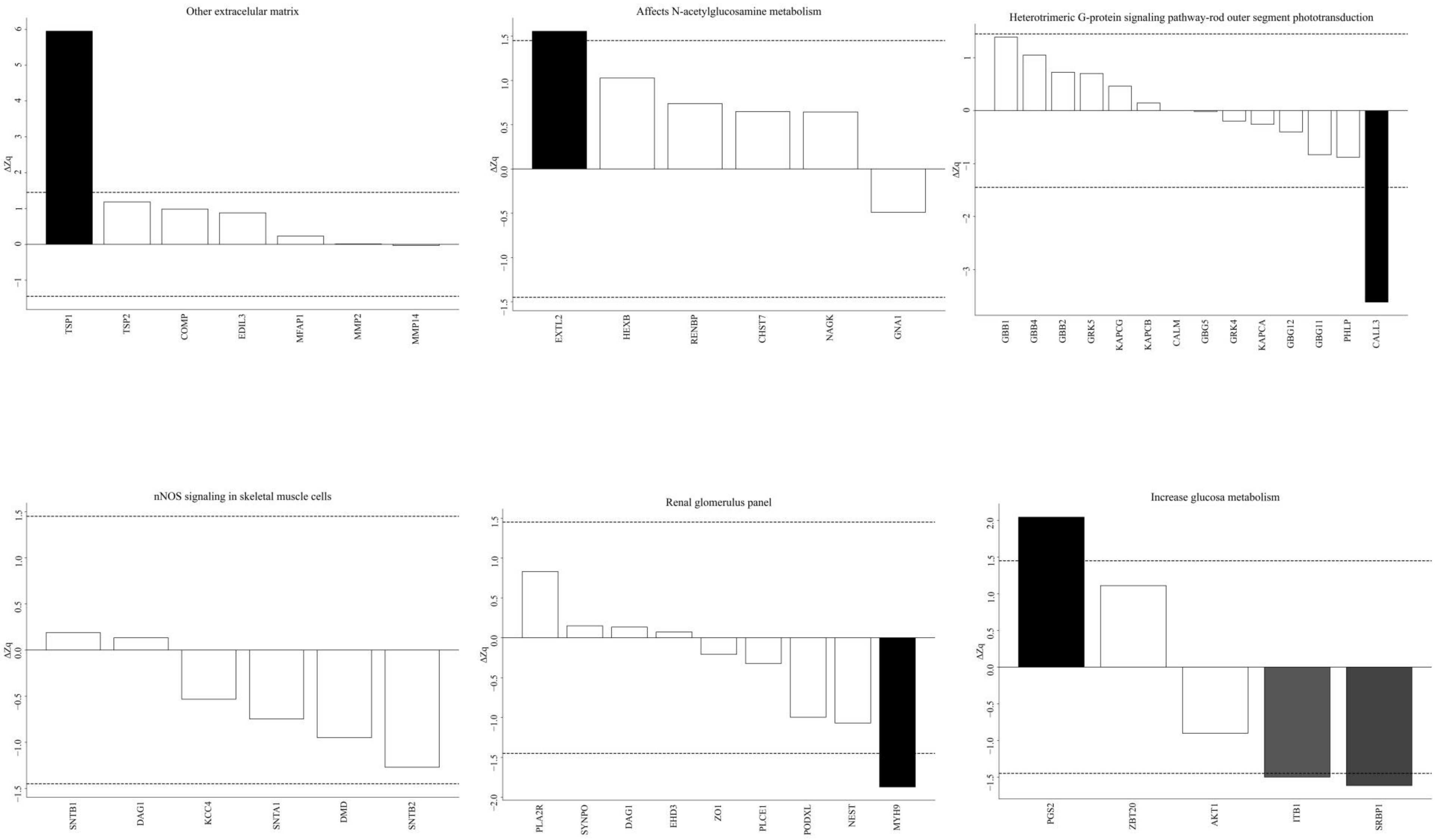

Supplemental Figure 1I

Others (2/2)

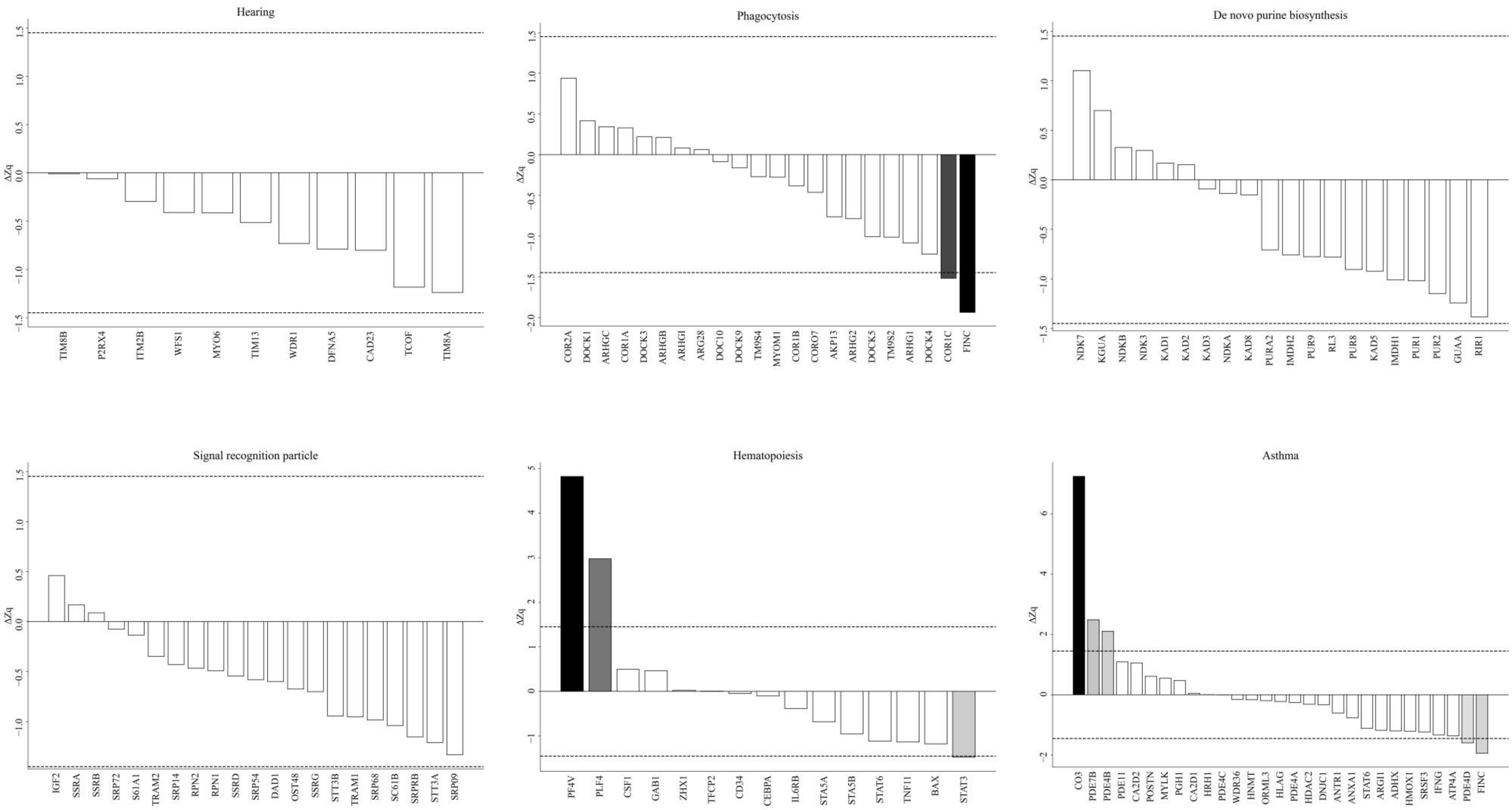

Supplemental Figure 1. Variation of specific proteins that contribute to the alteration of the different categories in EC-anaphylaxis. The graph represents the list of proteins identified in the different subcategories, their specific ΔZq. **(A)** Related to G protein, includes proteins from the GNAS complex locus and Rap1 complex subcategories. **(B)** Receptors, includes proteins from the subcategories of 5HT2 type receptor mediated signaling pathway, Histamine H2 receptor mediated signaling pathway, Thyrotropin-releasing hormone receptor signaling pathway and Tlr 1/2. **(C)** Cytoskeleton, includes proteins from the subcategories of Kinesin transport, TCR actin (T cell receptor), DRG1 complex, Actin binding motor protein and Myosin. **(D)** Cell Signaling, includes proteins from the JAK-STAT cascade, STAT1 complex, Cell proliferation, Cytotoxicity of leukocytes, Interferon and Interferon-mediated immunity subcategories. **(E)** Enzymes, includes proteins from the subcategories Glycosidase, Kinase inhibitor, Phospholipases, Lymphocyte-specific protein tyrosine kinase, tRNA ligase, Multisynthetase complex and Kinase maturation enzyme complex 1. **(F)** PTMs, includes proteins from the Ubiquitin-conjugate subcategories E2, Sumo1 complex and E3 ubiquitin ligase. **(G)** Nuclear - DNA, includes proteins from the subcategories of Set1A complex, CSA-POLIIa complex, TERF2-RAP1 complex, H2AX complex I, U5 snRNP complex, DDB2 complex, Other nucleic acid binding, TRBP containing complex, XRCC5 complex, Cell cycle: G2-M DNA damage checkpoint regulation, SNW1 complex, NCOA6-DNA-PK-Ku-PARP1 complex, DHX9-ADAR-vigilin-DNA-PK-Ku antigen complex, DNA repair and TLE1 corepressor complex. **(H and I)** Others, includes the proteins of the subcategories of Other extracellular matrix, Affects N-acetylglucosamine metabolism, Heterotrimeric G-protein signaling pathway-rod outer segment phototransduction, nNOS signaling in skeletal muscle cells, Renal glomerulus panel, Increase glucose metabolism , Hearing, Phagocytosis, De novo purine biosynthesis, Signal recognition particle, Hematopoiesis and Asthma.
